# Infectivity and antigenicity of pseudoviruses with high-frequency mutations of SARS-CoV-2 identified in Portugal

**DOI:** 10.1007/s00705-021-05327-0

**Published:** 2022-01-27

**Authors:** Hai-xin Wang, Li Zhang, Zi-teng Liang, Jian-hui Nie, Jia-jing Wu, Qian-qian Li, Ru-xia Ding, Yue Zhang, Guo-qing Chen, You-chun Wang, Hui-guo Wang, Wei-jin Huang

**Affiliations:** 1 Department of Pharmaceutical Engineering, College of life science and technology, Dalian University, Dalian, 116622 Liaoning China; 2 AIDS Department, Institute of Biological Products, China Food and Drug Testing Institute, Beijing, 102629 China

## Abstract

Severe acute respiratory syndrome coronavirus 2 (SARS-CoV-2) has had a major impact on global human health. During the spread of SARS-CoV-2, weakened host immunity and the use of vaccines with low efficacy may result in the development of more-virulent strains or strains with resistance to existing vaccines and antibodies. The prevalence of SARS-CoV-2 mutant strains differs between regions, and this variation may have an impact on the effectiveness of vaccines. In this study, an epidemiological investigation of SARS-CoV-2 in Portugal was performed, and the VSV-ΔG-G* pseudovirus system was used to construct 12 spike protein epidemic mutants, D614G, A222V+D614G, B.1.1.7, S477N+D614G, P1162R+D614G+A222V, D839Y+D614G, L176F+D614G, B.1.1.7+L216F, B.1.1.7+M740V, B.1.258, B.1.258+L1063F, and B.1.258+N751Y. The mutant pseudoviruses were used to infect four susceptible cell lines (Huh7, hACE2-293T-293T, Vero, and LLC-MK2) and 14 cell lines overexpressing ACE2 from different species. Mutant strains did not show increased infectivity or cross-species transmission. Neutralization activity against these pseudoviruses was evaluated using mouse serum and 11 monoclonal antibodies. The neutralizing activity of immunized mouse serum was not significantly reduced with the mutant strains, but the mutant strains from Portugal could evade nine of the 11 monoclonal antibodies tested. Neutralization resistance was mainly caused by the mutations S477N, N439K, and N501Y in the spike-receptor binding domain. These findings emphasize the importance of SARS-CoV-2 mutation tracking in different regions for epidemic prevention and control.

**Supplementary Information:**

The online version contains supplementary material available at 10.1007/s00705-021-05327-0.

## Introduction

Since the beginning of the coronavirus disease 2019 (COVID-19) pandemic, multiple virus variants have emerged that differ from the original virus by mutations in different genes. Some variants have been shown to possess altered biological properties that increase their transmissibility and/or ability to evade pre-existing immunity after convalescence or vaccination. As a result, some SARS-CoV-2 variants have rapidly expanded on a local or global level and challenged our efforts to control the pandemic through vaccination programs and containment strategies. To quickly respond to newly emerging variants that may become future drivers of the pandemic, it is important to constantly monitor SARS-CoV-2 diversity in different regions of the world and to identify virus variants with altered biological properties. The frequency of SARS-CoV-2 spike (S) protein mutations is high, and the neutralizing activity of some vaccines and monoclonal antibodies (mAbs) against mutant South African strains is low [1–3]. Mutant strains of the virus in the United Kingdom, the United States, Brazil, South Africa, and other regions have attracted widespread attention. However, few studies have focused on mutant strains in Portugal. In this study, virus strains containing the N501Y mutation in Portugal were traced. Initially discovered in December 2021, UK variants and other additional variants spread to Portugal. To further understand the biological activity of SARS-CoV-2 epidemic strains in Portugal, we used a pseudovirus system with a vesicular stomatitis virus (VSV) vector to simulate the circulating viruses and analyzed the effect of mutations on transmission, infectivity, and antigenicity.

## Materials and methods

### Reagents

The main reagents, cells, and monoclonal antibodies used in the study are shown in Table 1.

**Table 1:** Cell lines and reagents

Cell line	Source
HEK-293T	Our laboratory
Huh7	Our laboratory
Vero	Our laboratory
LLC-MK2	Our laboratory
293T-ACE2	Sino Biological
mAb	Source
03-1F9	Beijing Biocytogen Co., Ltd.
09-7B8	Beijing Biocytogen Co., Ltd.
09-4E5-1G2-2H10	Beijing Biocytogen Co., Ltd.
03-10D12-1C3	Beijing Biocytogen Co., Ltd.
03-10F9-1A2	Beijing Biocytogen Co., Ltd.
11D12-1	Beijing Biocytogen Co., Ltd.
05-9G11-1G1	Beijing Biocytogen Co., Ltd.
CB6	Laboratory of Jinghua Yan
X593	Laboratory of Xiaoliang Xie
HB27	Sino Biological
Reagent name	Source
Lipofectamine3000 Transfection Reagent	Invitrogen
Bright-Glo Fluorescence Detection Reagent (substrate)	Promega
PE anti-DYKDDDDK Tag Antibody	Biolegend
Hygromycin B	Gibco

### Mouse sera

The mouse sera used in this study were maintained in our laboratory. Expression plasmid constructs encoding the full-length SARS-CoV-2 spike protein or S1, S2, or RBD region alone were used to immunize BALB/c mice to obtain serum for the testing of mutant pseudoviruses. The BALB/c mice were divided into four groups: S1 (n = 10), S2 (n = 10), RBD (n = 10), and full-length S (n = 15). Serum samples from five mice were combined and labeled S1-1, S1-2, S2-1, S2-2, RBD-1, RBD-2, S-1, S-2, and S-3. The animal research protocol was approved by the Animal Welfare Ethical Review Committee of the National Institute of Food and Drug Control.

### Plasmid construction

Mammalian codon optimization was performed on the plasmid expressing SARS-CoV-2 spike protein (GenBank accession number: MN908947). This gene was then inserted into the eukaryotic expression vector pcDNA3.1 at the BamHI and XhoI sites and designated as WuHan-1.

A total of 14 plasmids for expression of the ACE2 receptor were constructed, including the ACE2 receptors of human (BAB40370.1), mink (QNC68911.1), dog (MT663955), cat (MT663959), pangolin (XP_017505746.1), pig (NP_001116542.1), rat (ABN80106.1), bat (KC881004.1), cow (NP_001019673.2), rabbit (MT663961), ferret (MT663957), sheep (XP_011961657.1), civet (AY881174.1), and monkey (MT663960). A FLAG tag (GACTACAGAGACGATGATAAG) was inserted at the 3' end. The 14 ACE2 sequences were codon-optimized and synthesized by General Biologicals Co., Ltd. (Taiwan). Each ACE2 sequence was inserted into the eukaryotic expression vector pRP[Exp]-EGFP-CMV at the BamHI and XhoI sites to obtain plasmids expressing the ACE2 proteins of different species.

### Mutant construction

Point mutations were introduced into the WuHan-1 plasmid to construct 19 mutants: D614G, A222V+D614G, B.1.1.7, S477N+D614G, P1162R+D614G+A222V, D839Y+D614G, L176F+D614G, B.1.1.7+L216F, B.1.1.7+M740V, B.1.258, B.1.258+L1063F, B.1.258+N751Y, S477N, D839Y, L176F, L216F+D614G, M740V+D614G, L1063F+D614G, and N751Y+D614G. The point mutations were introduced as described in our previous study [4, 5]. Briefly, PCR amplification was performed using the WuHan-1 plasmid as a template. The PCR product was digested overnight with DpnI (NEB) and used to transform competent *E. coli* DH5α cells. Then, the cells were spread onto plates and incubated overnight at 37°C. A single colony was selected and sequenced to confirm the successful generation of the mutation. Primers corresponding to the mutation sites are shown in Table 2.

**Table 2:** Primer sequences

Name	Sequence
VSV-P-F	ATGGATAATCTCACAAAAGTTCGTGAGTATCT
VSV-P-R	CTACAGAGAATATTTGACTCTCGCCTGATTGTACA
D614G-F	TGCTGTACCAGGGCGTGAATTGCACCGAGGT
D614G-R	ACCTCGGTGCAATTCACGCCCTGGTACAGCA
M740V-F	AGCGTGGACTGCACCgtgTACATCTGCGGCGACA
M740V-R	TGTCGCCGCAGATGTAcacGGTGCAGTCCACGCT
L216F-F	TCTGGTGAGAGACttcCCTCAGGGCTTCAGCGCCCT
L216F-R	AGGGCGCTGAAGCCCTGAGGgaaGTCTCTCACCAGA
N751Y-F	ACCGAGTGCAGCtacCTGCTGCTGCAGTACGG
N751Y-R	CCGTACTGCAGCAGCAGgtaGCTGCACTCGGT
L1063F-F	CGCTCCACATGGCGTGGTGTTCttcCACGTGACCT
L1063F-R	AGGTCACGTGgaaGAACACCACGCCATGTGGAGCG
P1162R-F	AGAATCACACCAGCcgaGACGTGGACCTCGGT
P1162R-R	ACCGAGGTCCACGTCtcgGCTGGTGTGATTCT
A222V-F	CCTCAGGGCTTCAGCGTGCTGGAGCCTCTGGTGGA
A222V-R	TCCACCAGAGGCTCCAGCACGCTGAAGCCCTGAGG
D839Y-F	TTCATCAAGCAGTACGGCtatTGCCTAGGTGATA
D839Y-R	TATCACCTAGGCAataGCCGTACTGCTTGATGAA
L176F-F	TACGTGAGCCAGCCTTTCttcATGGACCTGGA
L176F-R	TCCAGGTCCATgaaGAAAGGCTGGCTCACGTA
S477N-F	TACCAGGCCGGCAATACACCGTGTAATGGCGTGGA
S477N-R	TCCACGCCATTACACGGTGTATTGCCGGCCTGGTA
A570D-F	CAACAATTCGGCAGAGACATCGACGACACCACAGATGCTGTAAGAGAC
A570D-R	GTCTCTTACAGCATCTGTGGTGTCGTCGATGTCTCTGCCGAATTGTTG
D1118H-F	ACGAGCCTCAGATCATCACCACCCACAATACCTTCGTGAGCGGCAA
D1118H-R	TTGCCGCTCACGAAGGTATTGTGGGTGGTGATGATCTGAGGCTCGT
69-70del-F	CGTGACCTGGTTCCACGCCATCAGCGGCACCAATGGCACCAAGAGATTC
69-70del-R	GAATCTCTTGGTGCCATTGGTGCCGCTGATGGCGTGGAACCAGGTCACG
N501Y-F	AGAGCTACGGCTTCCAGCCTACCTACGGCGTGGGCTACCAGCCTTACAG
N501Y-R	CTGTAAGGCTGGTAGCCCACGCCGTAGGTAGGCTGGAAGCCGTAGCTCT
N501Y-F	AGAGCTACGGCTTCCAGCCTACCTACGGCGTGGGCTACCAGCCTTACAG
N501Y-R	CTGTAAGGCTGGTAGCCCACGCCGTAGGTAGGCTGGAAGCCGTAGCTCT
P681H-F	CTACCAGACCCAGACCAATAGCCACAGAAGAGCCAGAAGCGTGGCCAGCC
P681H-R	GGCTGGCCACGCTTCTGGCTCTTCTGTGGCTATTGGTCTGGGTCTGGTAG
S982A-F	TACTCAACGACATCCTGGCGAGACTGGACAAGGTGGAGGCCGA
S982A-R	TCGGCCTCCACCTTGTCCAGTCTCGCCAGGATGTCGTTGAGTA
T716I-F	CAATAATAGCATCGCCATCCCTATCAATTTCACCATCAGCGTGACCAC
T716I-R	GTGGTCACGCTGATGGTGAAATTGATAGGGATGGCGATGCTATTATTG
145del-F	GACCCTTTCCTGGGTGTTTATCATAAGAACAACAAGAGCTGGATGG
145del-R	CCATCCAGCTCTTGTTGTTCTTATGATAAACACCCAGGAAAGGGTC
N439K-F	CTGCGTGATCGCGTGGAACTCTAAGAACCTGGACTCGAAAGTTGGAGGC
N439K-R	GCCTCCAACTTTCGAGTCCAGGTTCTTAGAGTTCCACGCGATCACGCAG

### Preparation of ACE2-overexpressing cells

Cells expressing the ACE2 receptors of different species were prepared. Lipofectamine 3000 transfection reagent and 30 μg of receptor plasmid were used to transfect HEK-293T cells in T75 flasks, yielding ACE2 receptor-overexpressing cells. After culturing in the same medium that was used for HEK-293T cells at 37°C and 5% CO_2_ for 24 h, the expression of the labeled ACE2 gene on the cell surface was evaluated by flow cytometry. Approximately 1 × 10^6^ cells per tube were treated with 1 μg of PE-labeled anti-labeled antibody (biological preparation) per mL.

### Preparation of pseudoviruses

SARS-CoV-2 pseudoviruses with spike mutations were constructed as described previously [4, 5]. The day before transfection, the concentration of HEK-293T cells was adjusted to 5–7 × 10^5^ cells/mL, followed by incubation overnight at 37°C and 5% CO_2_. When the cells reached 70%–90% confluence, the culture medium was removed by aspiration, and the cells were infected with 15 mL of VSV-ΔG-G* pseudovirus with a concentration of 7 × 10^4^ TCID_50_/mL. At the same time, cells were transfected with 30 μg of the S protein expression plasmid using Lipofectamine 3000 and cultured in an incubator at 37°C and 5% CO_2_. After 4–6 h, the cell culture medium was discarded, the cells were washed twice with PBS containing 1% FBS, and 15 mL of fresh medium was added to the T75 cell culture flask, which was placed in a 37°C incubator with 5% CO_2_ for 24 h. The supernatant (containing SARS-CoV-2 pseudovirus) was then collected.

### Quantification of pseudoviruses

Pseudovirus RNA was extracted using a QIAamp Viral RNA Mini Kit (QIAGEN, Hilden, Germany). An RT-PCR kit (Invitrogen) was used to obtain viral DNA by reverse transcription. RT-PCR was performed using TBGreen premixed ExTaqII (Takara). The virus copy number was calculated using the P protein gene plasmid of VSV as a standard. Primer sequences are shown in Table 2.

### Detection of infection

Cells (3 × 10^4^/100 µL) were added to each well of a 96-well cell culture plate, followed by the addition of 100 µL of the pseudovirus. After 24 h of incubation at 37°C and 5% CO_2_, chemiluminescence was monitored.

The volume of the supernatant in each well was adjusted to 100 µL, followed by the addition of 100 µL of luciferase substrate and cell lysis buffer (PerkinElmer, Fremont, CA, USA). After 2 min, 150 µL of the lysate was transferred to an opaque 96-well plate. A PerkinElmer EnSight plate reader was used to detect the luminescence signal, and data were recorded as relative luminescence units (RLU). The virus copy number calculated as described above was used to convert the RLU value to the copy number.

### Neutralization test

Luciferase gene expression was measured to determine the inhibitory effect of mAbs and serum on pseudovirus entry. The sample was serially diluted by a factor of three (initial dilution, 30-fold), with a total of six dilutions, and 50 μL of the virus suspension was added to each well. Each 96-well plate included six virus control wells (no antibody/serum) and six cell control wells (no virus/antibody). The 96-well plate was incubated at 37°C for 1 h, followed by the addition of 3 × 10^4^ Huh7 cells (100µL) to each well. After incubation for 24 h at 37°C and 5% CO_2_, luminescence was measured. The Reed–Muench method was used to calculate the 50% effective concentration (EC_50_) [4].

### Immunization of mice

For protein immunization, mice were inoculated subcutaneously with purified SARS-CoV-2 RBD protein, S1 region peptide, or S2 region peptide and alum adjuvant (20 μg of protein), three times at 7-day intervals. Blood samples were collected 7 days after the third immunization. Ten mice were immunized with each protein, and serum samples from five mice in each group were pooled for analysis.

For plasmid immunization, mice were inoculated intramuscularly by electroporation with SARS-COV-2 S full-length plasmid (50 μg), three times at 7-day intervals. Blood samples were collected 7 days after the third immunization. A total of 15 mice were immunized, and serum samples from five mice were pooled for analysis.

## Results

### Tracking of SARS-CoV-2 spike protein amino acid polymorphisms in Portugal

**Fig. 1:**
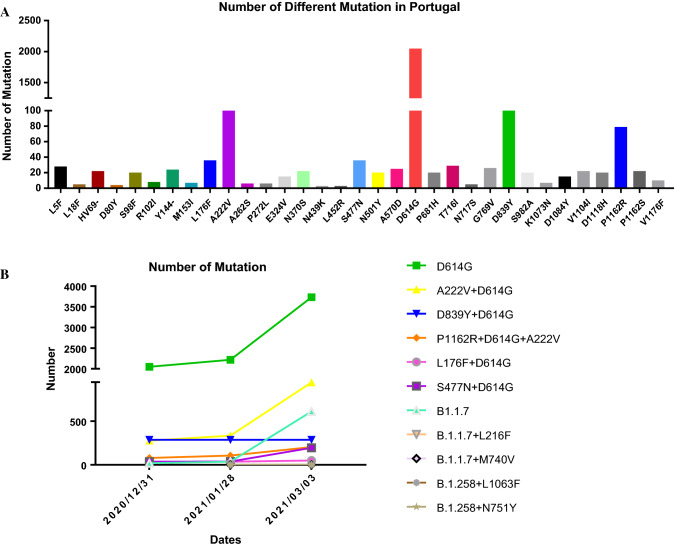
Mutation tracking of SARS-CoV-2 strains in Portugal. B.1.1.7 contains the spike (S) protein mutations A570D, D614G, D1118H, H69-V70del, N501Y, P681H, S982A, T716I, and Y145del. B.1.258 contains the S protein mutations D614G, N439K, and H69-V70del.

Using publicly available epidemiological surveillance data (https://covidcg.org/?tab=group), single non-synonymous substitutions in the spike protein of SARS-CoV-2 strains circulating in Portugal were tracked. Figure 1A shows the percentage of virus strains with polymorphisms among all strains in Portugal. The single amino acid substitutions with the highest frequencies were D614G (91.5%), D839Y (12.8%), A222V (12.3%), P1162R (3.5%), S477N (1.6%), and L176F (1.6%). Therefore, we screened six strains with single-site mutation frequencies greater than 1.5% and investigated combinations of mutations at these sites.

### Tracking of co-mutations in SARS-CoV-2 strains in Portugal

Using epidemiological data (https://www.epicov.org/epi3/frontend#1bd174), combined mutations in strains circulating in Portugal and the six most frequent single amino acid substitutions were tracked. Mutant strain B.1.1.7 spread rapidly in the United Kingdom in December and to various European countries, including Portugal. Among the mutant strains that appeared in Portugal in December, UK strain B.1.1.7 accounted for 73.1%; this strain possessed the L216F and M740V double mutation. At the same time, variant B.1.258, along with B.1.258+L1063F and B.1.258+N751Y, also appeared. Figure 1B shows the prevalence and changes in virus strains with a high frequency of naturally occurring joint mutations in Portugal. From December 31, 2020, to March 3, 2021, D614G remained the most frequent mutation (94.9%), followed by A222V+D614G (24.0%), B.1.1.7 (15.5%), D839Y+D614G (7.3%), P1162R+D614G+A222V (5.2%), S477N+D614G (5.0%), and L176F+D614G (1.3%). The frequencies of the variants D614G, A222V+D614G, B.1.1.7, S477N+D614G, and P1162R+D614G+A222V showed an increasing trend, with the increase in B.1.1.7 being second only to D614G. The frequencies of D839Y+D614G and L176F+D614G in Portugal showed a decreasing trend, and the prevalence of the B.1.1.7+L216F, B.1.1.7+M740V, B.1.258, B.1.258+L1063F, and B.1.258+N751Y variants did not increase.

### Infectivity analysis

#### Infectivity of mutant pseudoviruses in various cells

**Fig. 2:**
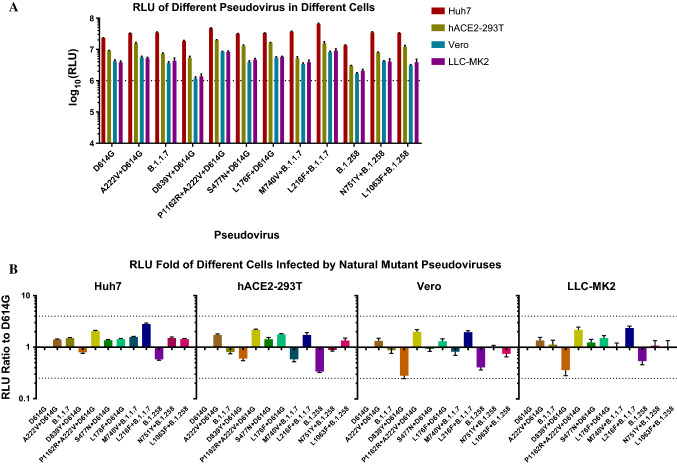
Infection of the four cell types with natural mutant pseudoviruses. (A) The 12 types of pseudoviruses were used to infect four susceptible cell lines (Huh7, Vero, hACE2-293T, and LLC-MK2), and RLU values were determined. All results are from three independent experiments (mean ± SD). In general, it was considered that a pseudovirus was successfully constructed when the RLU value reached at least10^4^ in the infected cells. The dotted line represents an RLU value of 10^6^, RLU values higher than10^6^ indicate high infectivity. (B) The infectivity of the mutant viruses tested in Huh7, Vero, hACE2-293T, and LLC-MK2 cells. The RLU value of the mutant infection was measured and compared with that of reference strain D614G. A fourfold difference was considered significant. This experiment was repeated six times (mean ± SD).

To evaluate the infectivity of natural variants of SARS-CoV-2 circulating in Portugal, we infected susceptible cells (Huh7, hACE2-293T, Vero, and LLC-MK2) with 12 mutant pseudoviruses. As shown in Figure 2A, infectivity (as evaluated using relative light unit [RLU] values) for all pseudoviruses was highest in Huh7 cells, followed by hACE2-293T cells, while Vero and LLC-MK2 cells were infected at relatively low levels. Figure 2B shows the infectivity of the mutants in descending order of frequency in Portugal. In all four types of cells, the RLU values for D839Y+D614G and B.1.258 were lower than those for D614G, and the infectivity of P1162R+D614G+A222V and L216F+B.1.1.7 was about two times higher than that of D614G. In general, the infectivity of the 11 natural mutants was similar to that of D614G (setting a four-fold increase as the threshold for a significant difference).

#### Infectivity of different mutant pseudoviruses in cells overexpressing the ACE2 receptor

SARS-CoV-2 infection occurs following binding of the S protein to the host cell ACE2 receptor. To evaluate the infectivity of the natural mutant strains circulating in Portugal, we infected HEK-293T cells overexpressing the ACE2 receptor with 12 strains containing natural mutations and determined the RLU values of the infected cells.

First, we constructed 14 cell lines expressing the ACE2 receptors of different species. After transient transfection, flow cytometry was used to detect the expression of ACE2 in each cell line. We used untreated HEK-293T cells as a negative control. The rate of ACE2 protein expression was 20.8%–43.8%, with an average of 32.3%.

**Fig. 3:**
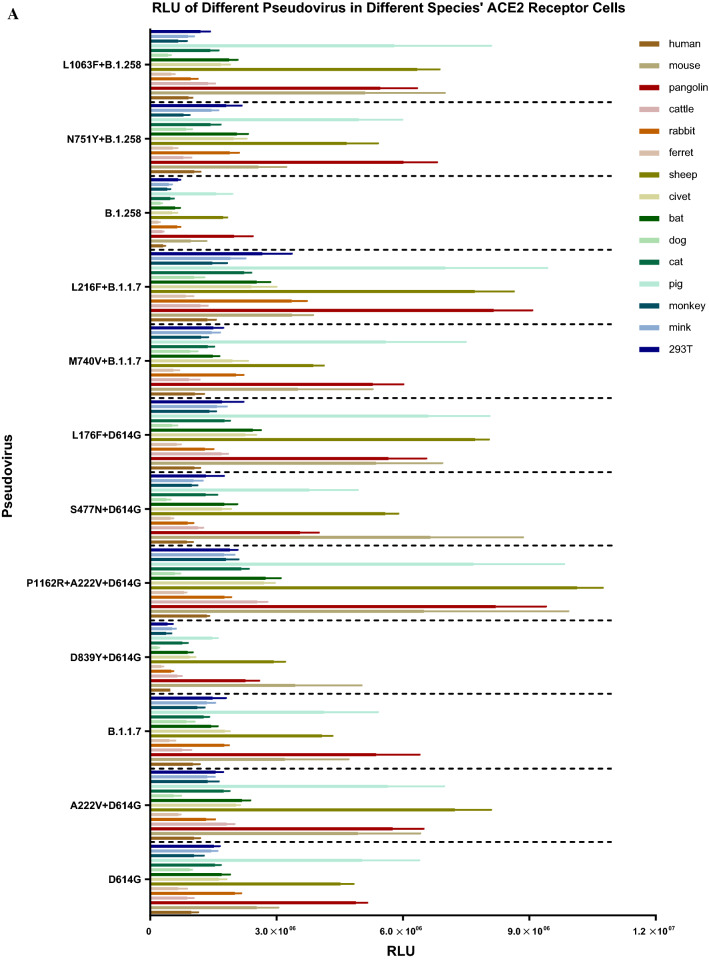

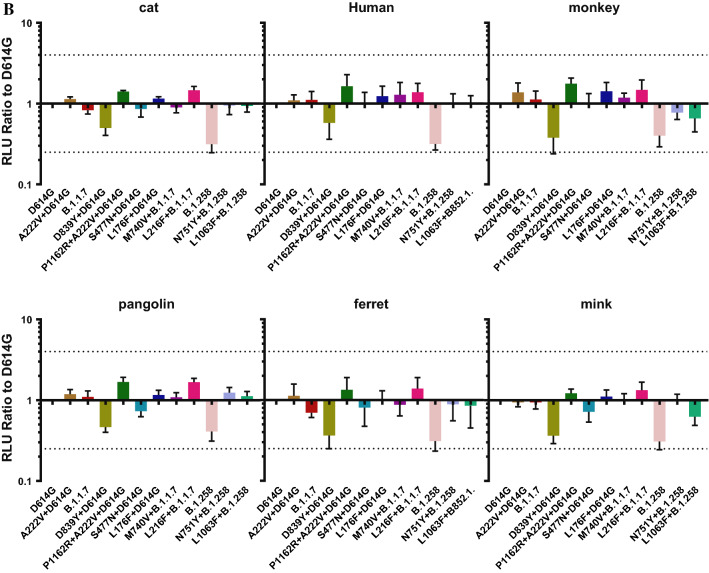

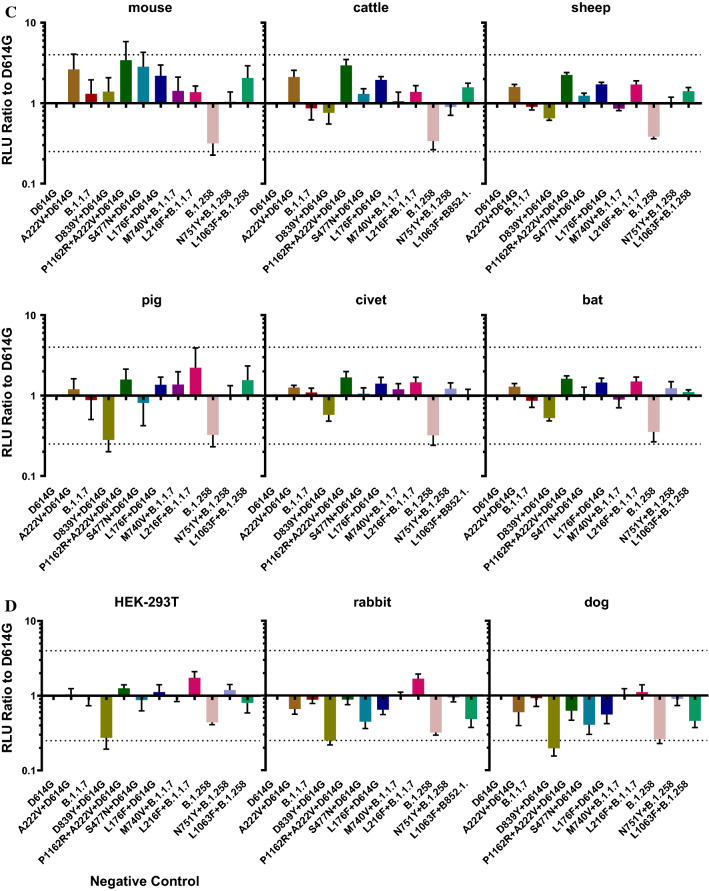
Pseudovirus infectivity in cells overexpressing ACE2 receptor from different taxa. The figure shows the infectivity (RLU values) of the 12 pseudoviruses in 14 ACE2-overexpressing cell lines from different species and HEK-293T (negative control) cells. (A) RLU values of the 12 pseudoviruses in the 15 cell lines, indicating the susceptibility of recipient cells from different species to different pseudoviruses. (B-D) Comparison of the RLU values of the other 11 pseudoviruses to that of D614G. Dotted lines at *x* = 4 represent fourfold higher infectivity that the standard, and those at *y* = 0.25 represent fourfold lower infectivity than the standard. The experiment was repeated four to six times (mean ± SD).

We then infected these 14 cell lines overexpressing ACE2 receptors with 12 pseudoviruses containing natural mutations. Figure 3 shows that in cells expressing ACE2 receptors of different species, the infectivity of the individual mutant pseudovirus did not differ by more than fourfold from that of D614G. However, all pseudoviruses constructed in this study were more infectious in 293T cells overexpressing mouse ACE2, pig ACE2, pangolin ACE2, and cattle ACE2 receptors than in other cells. The infectivity of all pseudoviruses was generally low in cells overexpressing rabbit ACE2 and dog ACE2 receptors. The experimental results suggest that SARS-CoV-2 presents a risk of trans-species infection.

### Neutralizing activity

#### Detection of neutralizing antibodies in serum after immunization

**Fig. 4:**
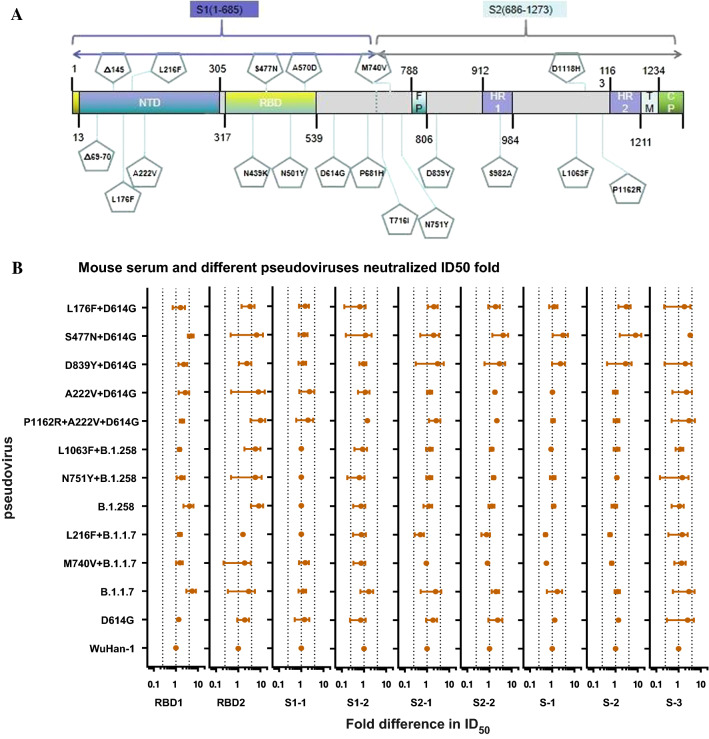
Neutralization activity of mouse serum with different pseudoviruses. (A) Distribution of SARS-CoV-2 mutation sites. (B) RBD1 and RBD2 represent the two groups of mice immunized with peptides corresponding to the SARS-CoV-2 RBD. S1-1 and S1-2 represent the two groups of mice immunized with peptides corresponding to SARS-CoV-2 S1. S2-1 and S2-2 represent the two groups of mice immunized with peptides corresponding to SARS-CoV-2 S2. S1, S2, and S3 represent three groups of mice immunized with the SARS-CoV-2 S full-length DNA plasmid. Dotted lines at *x* = 0.25 represent a fourfold decrease in neutralization activity compared to D614G, and those at *x* = 4 represent a fourfold increase in neutralization activity. The experiment was repeated four to six times (mean ± SD).

The distribution of the 12 natural mutations in the SARS-CoV-2 S protein in Portugal that were evaluated in the study is shown in Figure 4A. Currently, most mAbs are directed against the receptor binding domain (RBD) of the SARS-CoV-2 S protein. Therefore, the impact of the N439K, S477N, N501Y, and A570D mutations on protection by mAbs was evaluated. The effects of mutations at other sites on the transmission and infectivity of the virus warrant further study in the future.

First, we synthesized different segments of SARS-CoV-2 S peptides, including RBD, S1, and S2, and used these peptides as a vaccine to immunize BALB/c mice to obtain post-immunization serum. We also used the SARS-CoV-2 S full-length plasmid as a DNA vaccine to immunize BALB/c mice. We used these sera and 12 pseudoviruses to evaluate the protective effects of neutralizing antibodies (Fig. 4B). Using the neutralization ID_50_ (50% infective dose) titers for the D614G pseudovirus and different mouse sera for reference, the neutralization activity was evaluated for the other 11 pseudoviruses. The serum neutralized 11 pseudoviruses with no immune escape detected (defined as a fourfold reduction in the ID_50_ value compared with that for D614G).

### Monoclonal antibody neutralization activity

To identify specific sites affecting the antigenicity of the viruses circulating in Portugal, we constructed seven pseudoviruses: S477N, D839Y, L176F, L216F+D614G, M740V+D614G, L1063F+D614G, and N751Y+D614G. All pseudoviruses constructed in this study were treated with 11 mAbs. The IC_50_ values for the 11 mAbs were similar (i.e., approximately 0.03–0.18 μg/mL) and showed no significant differences (*P* > 0.05).

**Fig. 5:**
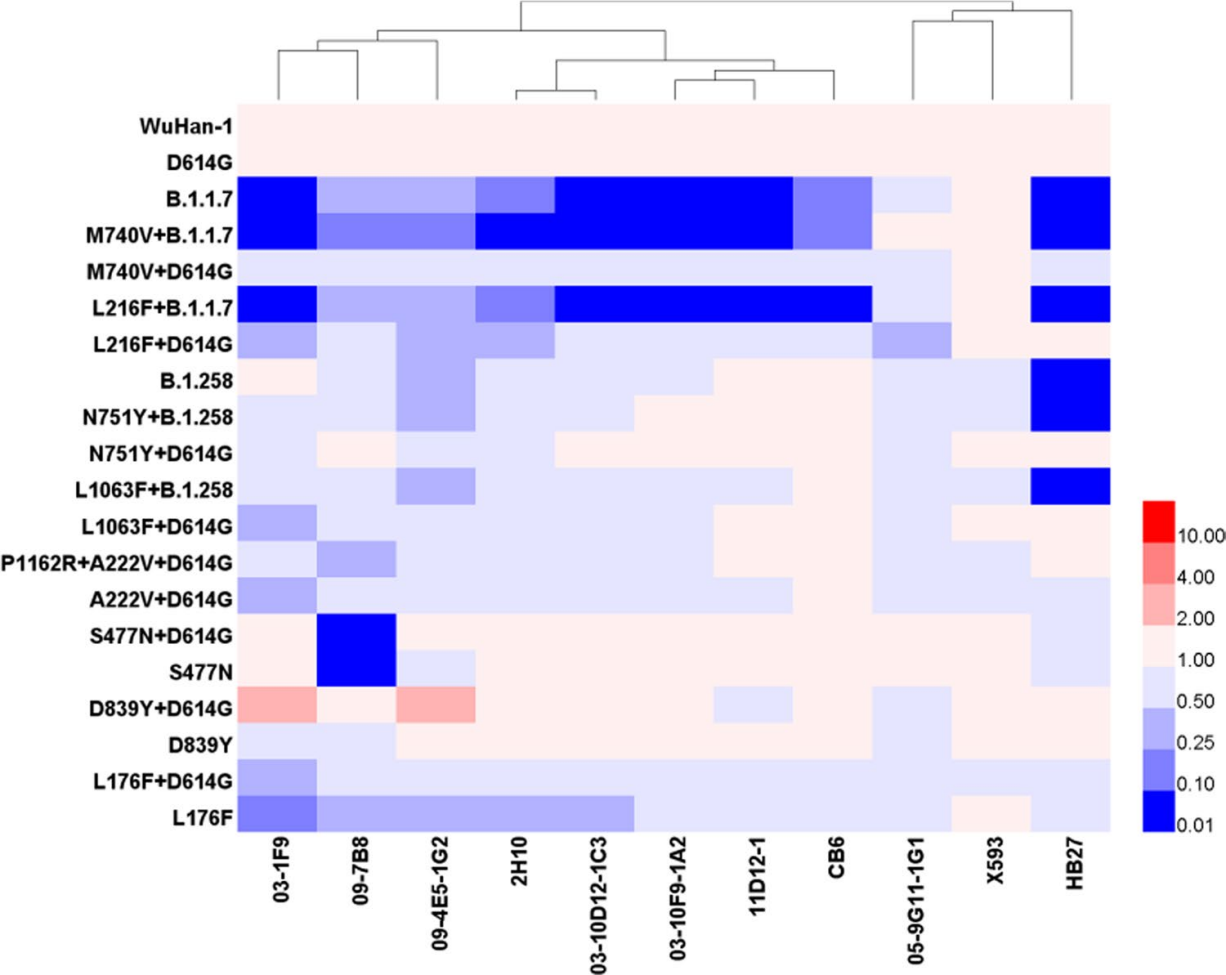
Neutralization effect of mAbs against different pseudoviruses. The neutralization ID_50_ value for D614G was used as the standard, and neutralization ID_50_ values were calculated for the different mutant strains of the pseudoviruses and the 11 mAbs. A significant difference was defined as a fourfold difference from the standard. Blue represents a decrease in neutralizing activity, and red represents an increase in neutralizing activity.

As shown in Figure 5, the epidemic strain S477N+D614G (accounting for 5.0% of strains) and its corresponding single-site mutant strain isolated in Portugal can evade mAb 09-7B8. Immune escape may therefore be attributed to the S477N mutation (using D614G as the standard and a reduction in neutralizing activity of >4 times as the threshold for immune escape). We also found that the UK epidemic strain B.1.1.7 and its variants can escape mAbs 03-1F9, 2H10, 03-10D12- 1C3, 03-10F9-1A2, 11D12-1, CB6, and HB27, but M740V+D614G and L216F+D614G cannot. Therefore, immune escape may mainly be caused by a mutation in B.1.1.7. In addition to escape from mAb HB27 by the UK strain, immune escape was observed for B.1.258 and its variants. Compared with the neutralizing effects of L1063F+D614G and N751Y+D614G, immune escape may be due to a mutation in B.1.258.

## Discussion

Coronaviruses have the longest genomes among RNA viruses and are highly prone to mutations. Because of the global health impact of SARS-CoV-2, real-time monitoring of SARS-CoV-2 mutations is essential. The need for biosafety level three laboratories, the infection risk for operators, and the shortage of experimental equipment have limited the development of SARS-CoV-2 vaccines. In this study, the VSV system was used to construct pseudoviruses harboring high-frequency SARS-CoV-2 mutations circulating in Portugal, including D614G, A222V+D614G, B.1.1.7, D839Y+D614G, P1162R+D614G+A222V, S477N+D614G, and L176F+D614G, as well as mutations found in UK strains, including B.1.1.7+L216F, B.1.1.7+M740V, B.1.258, B.1.258+L1063F, and B.1.258+N751Y. The infectivity and antigenicity of the constructed pseudoviruses were analyzed.

There was no significant difference in infectivity between the Portuguese high-frequency mutants and the D614G mutant pseudovirus (a ratio >4 was defined as a significant difference). Previous studies have shown that compared with D614G, the infectivity of B.1.1.7 in cells containing the hACE2 receptor showed no significant difference, which is consistent with the results of this study [6]. In the current study, the infectivity of B.1.1.7 did not change significantly after addition of the M740V or L216F mutation. When cells overexpressing receptors of different species were infected with the constructed pseudoviruses, the infectivity of all mutant pseudoviruses was higher in cells harboring the mouse ACE2 receptor. This is consistent with the results of Gu and colleagues [7].

During the ongoing pandemic, the emergence of various SARS-CoV-2 mutants has become a major issue. Mutants may have a stronger transmission capacity or the ability to evade neutralizing monoclonal and polyclonal antibodies. This may reduce the protective effects of vaccines or neutralizing mAbs developed based on the original genotypes. Nie and colleagues found that the neutralization effect of an antiserum against the South African epidemic strain was reduced against the 501Y.V2 variant [4]. We speculate that established vaccines prepared against WuHan-01 may have reduced neutralizing activity against current strains circulating in Portugal. Because the mutation sites in the epidemic strains circulating in Portugal are located in different regions of the spike protein, we used the SARS-CoV-2 spike full-length plasmid and RBD, S1, and S2 peptides to immunize mice, and serum was collected. In this analysis, serum neutralizing activity did not differ among the pseudoviruses.

Interestingly, for the majority of high-frequency mutant viruses circulating in Portugal (i.e., A222V+D614G, B.1.1.7, D839Y+D614G, P1162R+D614G+A222V, S477N+D614G, and L176F+D614G, but not B.1.1.7 and S477N+D614G), immune escape from the mAbs used in this study was not observed. The antigenicity of B.1.1.7 and derived viruses with combined mutations (M740V+B.1.1.7 and L216F+B.1.1.7), and B.1.258 and derived viruses with combined mutations (B.1.258+L1063F and B.1.258+N751Y) differed. The mutant viruses exhibited immune escape with MAbs 03-1F9, 2H10, 03-10D12-1C3, 03-10F9-1A2, 11D12-1, CB6, and HB27. The epitopes of 03-10D12-1C3 and CB6 include amino acid 501 [8]. The pseudoviruses that exhibited immune escape all contained the N501Y mutation, and the results of this experiment were consistent with the expected results. The epitopes for the other mAbs are still unknown, but we speculate that the mutation at position 501 may be the cause of immune escape. The neutralizing activity of various RBD mAbs was significantly reduced. Previous studies have shown that the N501Y mutation affects the antigenicity of the S protein. Because this site is located in the RBD region, most mAbs are directed against the RBD. Therefore, pseudoviruses containing the N501Y mutation will undergo immune escape or a decrease in the protective effects of the mAb will be observed [6, 9, 10]. The mutant virus B.1.258 (containing the mutations N439K and Δ69-70Del) also exhibits immune escape from the mAb HB27 [11]. Although the specific epitope of HB27 is unknown, it is predicted to be concentrated within the RBD region [12], potentially explaining the observed immune escape by S477N+D614G, and the corresponding single point mutant S477N showed immune escape from mAb 09-7B8. Nie and coworkers found that the 09-7B8 epitope was located in the RBD region [12], which is consistent with the results of this study. At present, the S477N mutation accounts for 5.0% of mutant strains circulating in Portugal. With the exception of X593 and 05-9G11-1G1, immune escape by individual mutant strains was observed with all of the mAbs evaluated in this study. To ensure the protective effects of vaccines, cocktail therapies are recommended against SARS-CoV-2 mutant strains [13, 14]. We should also continue to monitor SARS-CoV-2 mutations in different regions in real time, and select mAbs for the corresponding mutations to ensure immune protection according to the epidemic situation regarding mutant strains in different regions. It is vital that epidemic prevention and control measures are adjusted in real time.

Our laboratory has studied mutant strains from South Africa, the United Kingdom, and Brazil, and this study extends our analysis to mutant strains from Portugal. The results are important for SARS-CoV-2 mutation tracking and provide data for SARS-CoV-2 epidemic prevention and control.

## Supplementary Information

Below is the link to the electronic supplementary material.

Supplementary file1 (DOC 37 kb)

Supplementary file2 (DOCX 14 kb)

## References

[1] Cele S, Gazy I, Jackson L (2021). Escape of SARS-CoV-2 501Y.V2 from neutralization by convalescent plasma. Nature.

[2] Shen X, Tang H, Pajon R (2021). Neutralization of SARS-CoV-2 Variants B.1.429 and B.1.351. N Engl J Med.

[3] Andreano E, Piccini G, Licastro D (2020). SARS-CoV-2 escape in vitro from a highly neutralizing COVID-19 convalescent plasma. bioRxiv.

[4] Nie J, Li Q, Wu J (2020). Quantification of SARS-CoV-2 neutralizing antibody by a pseudotyped virus-based assay. Nat Protoc.

[5] Nie J, Li Q, Wu J (2020). Establishment and validation of a pseudovirus neutralization assay for SARS-CoV-2. Emerg Microbes Infect.

[6] Li Q, Nie J, Wu J (2021). (2020) SARS-CoV-2 501YV2 variants lack higher infectivity but do have immune escape. Cell.

[7] Gu H, Chen Q, Yang G (2020). Adaptation of SARS-CoV-2 in BALB/c mice for testing vaccine efficacy. Science.

[8] Huang Y, Sun H, Yu H (2020). Neutralizing antibodies against SARS-CoV-2: current understanding, challenge and perspective. Antibody Therap.

[9] Chen RE, Zhang X, Case JB (2021). Resistance of SARS-CoV-2 variants to neutralization by monoclonal and serumderived polyclonal antibodies. Nat Med.

[10] Wang P, Nair MS, Liu L (2021). Antibody resistance of SARS-CoV-2 variants B.1.351 and B.1.17. Nature.

[11] Thomson EC, Rosen LE, Shepherd JG (2021). Circulating SARS-CoV-2 spike N439K variants maintain fitness while evading antibody-mediated immunity. Cell.

[12] Nie J, Xie J, Liu S (2021). Three epitope-distinct human antibodies from RenMab mice neutralize SARS-CoV-2 and cooperatively minimize the escape of mutants. Cell Discov..

[13] Wang N, Sun Y, Feng R (2021). Structure-based development of human antibody cocktails against SARS-CoV-2. Cell Res.

[14] Sun Y, Wang L, Feng R (2021). Structure-based development of three- and four-antibody cocktails against SARS-CoV-2 via multiple mechanisms. Cell Res.

