# Supplementary material for: Infectivity and antigenicity of pseudoviruses with high-frequency mutations of SARS-CoV-2 identified in Portugal
Source: Arch Virol. 2022 Jan 27;167(2):459–70. doi: 10.1007/s00705-021-05327-0 (PMC8791682; doi:10.1007/s00705-021-05327-0)
Supplement: Supplementary file 1 — Supplementary file1 (DOC 37 kb) [file 705_2021_5327_MOESM1_ESM.doc]

Table S1 Cell lines and reagents

| Cell lines | Source |
| --- | --- |
| HEK-293T | Our laboratory |
| Huh7 | Our laboratory |
| Vero | Our laboratory |
| LLC-MK2 | Our laboratory |
| 293T-ACE2 | Sino Biological |
| mAb | Source |
| 03-1F9 | Beijing Biocytogen Co., Ltd. |
| 09-7B8 | Beijing Biocytogen Co., Ltd. |
| 09-4E5-1G2-2H10 | Beijing Biocytogen Co., Ltd. |
| 03-10D12-1C3 | Beijing Biocytogen Co., Ltd. |
| 03-10F9-1A2 | Beijing Biocytogen Co., Ltd. |
| 11D12-1 | Beijing Biocytogen Co., Ltd. |
| 05-9G11-1G1 | Beijing Biocytogen Co., Ltd. |
| CB6 | Laboratory of Jinghua Yan |
| X593 | Laboratory of Xiaoliang Xie |
| HB27 | Sino Biological |
| Reagent name | Source |
| Lipofectamine3000 Transfection Reagent | Invitrogen |
| Bright-Glo Fluorescence Detection Reagent (substrate) | Promega |
| PE anti-DYKDDDDK Tag Antibody | Biolegend |
| Hygromycin B | Gibco |
