# Supplementary material for: Infectivity and antigenicity of pseudoviruses with high-frequency mutations of SARS-CoV-2 identified in Portugal
Source: Arch Virol. 2022 Jan 27;167(2):459–70. doi: 10.1007/s00705-021-05327-0 (PMC8791682; doi:10.1007/s00705-021-05327-0)
Supplement: Supplementary file 2 — Supplementary file2 (DOCX 14 kb) [file 705_2021_5327_MOESM2_ESM.docx]

|  | Table 2. Primer sequences |
| --- | --- |
| Name | Sequence |
| VSV-P-F | ATGGATAATCTCACAAAAGTTCGTGAGTATCT |
| VSV-P-R | CTACAGAGAATATTTGACTCTCGCCTGATTGTACA |
| D614G-F | TGCTGTACCAGGGCGTGAATTGCACCGAGGT |
| D614G-R | ACCTCGGTGCAATTCACGCCCTGGTACAGCA |
| M740V-F | AGCGTGGACTGCACCgtgTACATCTGCGGCGACA |
| M740V-R | TGTCGCCGCAGATGTAcacGGTGCAGTCCACGCT |
| L216F-F | TCTGGTGAGAGACttcCCTCAGGGCTTCAGCGCCCT |
| L216F-R | AGGGCGCTGAAGCCCTGAGGgaaGTCTCTCACCAGA |
| N751Y-F | ACCGAGTGCAGCtacCTGCTGCTGCAGTACGG |
| N751Y-R | CCGTACTGCAGCAGCAGgtaGCTGCACTCGGT |
| L1063F-F | CGCTCCACATGGCGTGGTGTTCttcCACGTGACCT |
| L1063F-R | AGGTCACGTGgaaGAACACCACGCCATGTGGAGCG |
| P1162R-F | AGAATCACACCAGCcgaGACGTGGACCTCGGT |
| P1162R-R | ACCGAGGTCCACGTCtcgGCTGGTGTGATTCT |
| A222V-F | CCTCAGGGCTTCAGCGTGCTGGAGCCTCTGGTGGA |
| A222V-R | TCCACCAGAGGCTCCAGCACGCTGAAGCCCTGAGG |
| D839Y-F | TTCATCAAGCAGTACGGCtatTGCCTAGGTGATA |
| D839Y-R | TATCACCTAGGCAataGCCGTACTGCTTGATGAA |
| L176F-F | TACGTGAGCCAGCCTTTCttcATGGACCTGGA |
| L176F-R | TCCAGGTCCATgaaGAAAGGCTGGCTCACGTA |
| S477N-F | TACCAGGCCGGCAATACACCGTGTAATGGCGTGGA |
| S477N-R | TCCACGCCATTACACGGTGTATTGCCGGCCTGGTA |
| A570D-F | CAACAATTCGGCAGAGACATCGACGACACCACAGATGCTGTAAGAGAC |
| A570D-R | GTCTCTTACAGCATCTGTGGTGTCGTCGATGTCTCTGCCGAATTGTTG |
| D1118H-F | ACGAGCCTCAGATCATCACCACCCACAATACCTTCGTGAGCGGCAA |
| D1118H-R | TTGCCGCTCACGAAGGTATTGTGGGTGGTGATGATCTGAGGCTCGT |
| 69-70del-F | CGTGACCTGGTTCCACGCCATCAGCGGCACCAATGGCACCAAGAGATTC |
| 69-70del-R | GAATCTCTTGGTGCCATTGGTGCCGCTGATGGCGTGGAACCAGGTCACG |
| N501Y-F | AGAGCTACGGCTTCCAGCCTACCTACGGCGTGGGCTACCAGCCTTACAG |
| N501Y-R | CTGTAAGGCTGGTAGCCCACGCCGTAGGTAGGCTGGAAGCCGTAGCTCT |
| P681H-F | CTACCAGACCCAGACCAATAGCCACAGAAGAGCCAGAAGCGTGGCCAGCC |
| P681H-R | GGCTGGCCACGCTTCTGGCTCTTCTGTGGCTATTGGTCTGGGTCTGGTAG |
| S982A-F | TACTCAACGACATCCTGGCGAGACTGGACAAGGTGGAGGCCGA |
| S982A-R | TCGGCCTCCACCTTGTCCAGTCTCGCCAGGATGTCGTTGAGTA |
| T716I-F | CAATAATAGCATCGCCATCCCTATCAATTTCACCATCAGCGTGACCAC |
| T716I-R | GTGGTCACGCTGATGGTGAAATTGATAGGGATGGCGATGCTATTATTG |
| 145del-F | GACCCTTTCCTGGGTGTTTATCATAAGAACAACAAGAGCTGGATGG |
| 145del-R | CCATCCAGCTCTTGTTGTTCTTATGATAAACACCCAGGAAAGGGTC |
| N439K-F | CTGCGTGATCGCGTGGAACTCTAAGAACCTGGACTCGAAAGTTGGAGGC |
| N439K-R | GCCTCCAACTTTCGAGTCCAGGTTCTTAGAGTTCCACGCGATCACGCAG |
